# Hybrid ultrasound and single wavelength optoacoustic imaging reveals muscle degeneration in peripheral artery disease

**DOI:** 10.1016/j.pacs.2023.100579

**Published:** 2023-12-02

**Authors:** Anna P. Träger, Josefine S. Günther, Roman Raming, Lars-Philip Paulus, Werner Lang, Alexander Meyer, Julius Kempf, Milenko Caranovic, Yi Li, Alexandra L. Wagner, Lina Tan, Vera Danko, Regina Trollmann, Joachim Woelfle, Daniel Klett, Markus F. Neurath, Adrian P. Regensburger, Markus Eckstein, Wolfgang Uter, Michael Uder, Yvonne Herrmann, Maximilian J. Waldner, Ferdinand Knieling, Ulrich Rother

**Affiliations:** a Department of Vascular Surgery, University Hospital Erlangen, Friedrich-Alexander-Universität Erlangen-Nürnberg (FAU), Krankenhausstraße 12, D-91054 Erlangen, Germany; b Department of Medicine 1, University Hospital Erlangen, Friedrich-Alexander-Universität Erlangen-Nürnberg (FAU), Ulmenweg 18, D-91054 Erlangen, Germany; c Department of Pediatrics and Adolescent Medicine, University Hospital Erlangen, Friedrich-Alexander-Universität Erlangen-Nuremberg (FAU), Loschgestraße 15, D-91054 Erlangen, Germany; d Department of Pathology, University Hospital Erlangen, Friedrich-Alexander-Universität Erlangen-Nürnberg (FAU), Krankenhausstrasse 8-10, D-91054 Erlangen, Germany; e Department of Medical Informatics, Biometry and Epidemiology, Friedrich-Alexander-Universität Erlangen-Nürrnberg (FAU), Waldstraße 6, D-91054 Erlangen, Germany; f Faculty of Medicine, Friedrich-Alexander-Universität Erlangen-Nürnberg (FAU), Krankenhausstraße 12, D-91054 Erlangen, Germany; g Deutsches Zentrum für Immuntherapie (DZI), University Hospital Erlangen, Ulmenweg 18, D-91054 Erlangen, Germany; h Erlangen Graduate School in Advanced Optical Technologies (SAOT), Friedrich-Alexander-Universität Erlangen-Nürnberg, Paul-Gordan-Straße 6, D-91052 Erlangen, Germany; i Institute of Radiology, University Hospital Erlangen, Friedrich-Alexander, Universität Erlangen-Nürnberg (FAU), Maximiliansplatz 1, D-91054 Erlangen, Germany; j Department of Pediatric Cardiology, University Hospital Erlangen, Friedrich-Alexander-Universität Erlangen-Nürnberg (FAU), Loschgestraße 15, D-91054 Erlangen, Germany

**Keywords:** Peripheral artery disease, Photoacoustics, Optoacoustics, Muscle imaging, Muscle degeneration

## Abstract

Peripheral arterial disease (PAD) leads to chronic vascular occlusion and results in end organ damage in critically perfused limbs. There are currently no clinical methods available to determine the muscular damage induced by chronic mal-perfusion. This monocentric prospective cross-sectional study investigated n = 193 adults, healthy to severe PAD, in order to quantify the degree of calf muscle degeneration caused by PAD using a non-invasive hybrid ultrasound and single wavelength optoacoustic imaging (US/SWL-OAI) approach. While US provides morphologic information, SWL-OAI visualizes the absorption of pulsed laser light and the resulting sound waves from molecules undergoing thermoelastic expansion. US/SWL-OAI was compared to multispectral data, clinical disease severity, angiographic findings, phantom experiments, and histological examinations from calf muscle biopsies. We were able to show that synergistic use of US/SWL-OAI is most likely to map clinical degeneration of the muscle and progressive PAD.

## 1 Introduction

With a global prevalence of 5,56% from 2011 to 2019, peripheral artery disease (PAD) is the third most common manifestation of arteriosclerosis, affecting an estimated 236,62 million people ≥ 25 years worldwide [1], [2], [3]. The disease is characterized by chronic insufficient blood supply in the lower limbs leading to pain and reduction of walking distance [4]. The symptoms derive from mal-perfusion of the muscle. If present chronically, this leads to structural changes in the musculature [5], [6], which include decreased fiber diameters, deterioration and oxidative fiber damage as well as mitochondrial dysfunction and intramuscular collagen matrix accumulation [5], [7], [8], [9], [10]. In advanced PAD, muscles appear with a mixed pattern of fragmented and enlarged fibers, signs of necrosis, and increased collagen content compared to healthy skeletal muscles [7], [11].

Current outcome measures in PAD mainly focus on macrocirculation and skin microperfusion, and therefore hardly represent muscle function. However, muscular damage may occur already at early stages [12]. To determine the degree of muscle degeneration as a result of chronic mal-perfusion, the only currently available method for quantification of this damage is biopsy sampling with histological analysis [13]. While approaches in regenerative medicine aiming to prevent or slow muscle degeneration offer tremendous benefit [14], [15], there is still an unmet clinical need to establish noninvasive and easy-applicable imaging modalities to quantify muscle degeneration.

In contrast to standard ultrasound (US), using B-mode techniques, optoacoustic imaging (OAI) offers the ability to visualize tissue absorbers non-invasively by using pulsed laser light excitation and ultrasound detection [16], [17]. The use of multiple wavelengths enable the visualization of different tissue absorbers directly by their unique absorption and spectral properties [17], [18], [19], [20], [21], [22], [23], [24], [25], [26], [27], [28], [29]. Consequently, multispectral optoacoustic imaging derived oxygenated hemoglobin has been shown to provide a potential biomarker for staging PAD [30]. While identification of spectral moieties within multispectral images requires dedicated hardware and coupling with post-processing to perform spectral unmixing, single wavelength signals may reveal unique morphological features of PAD [31]. In particular, the use of simple parameter and thus simple hardware configurations could significantly accelerate clinical translation. Especially 800 nm, the isosbestic point of hemo- and myoglobin, might be ideal to map the tissue properties more independently of the oxygen saturation present [32].

In this regard, we aimed to use directly available B-mode ultrasound and single wavelength optoacoustic imaging (SWL-OAI) 800 nm signals and compared them to an available parallel acquired multispectral imaging dataset. The imaging findings were correlated to clinical disease severity, and angiographic findings. Moreover, we further validated the origin of OAI signals in phantom experiments and histological examinations from calf muscle biopsies. Last, we investigated the susceptibility of SWL-OAI measurements to dynamic saturation fluctuations in a clinical context.

## 2 Results

### 2.1 Study sample

**Fig. 1:**
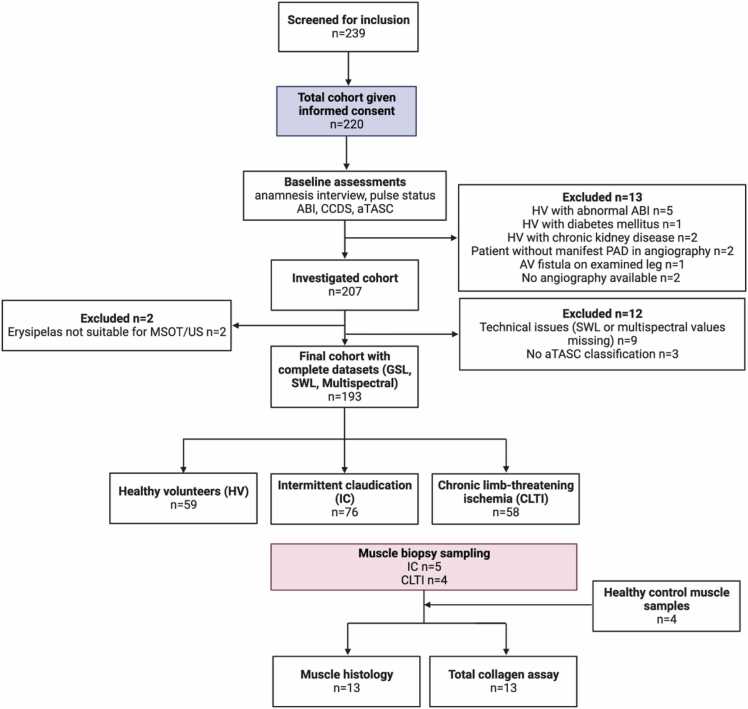
Flow of enrolled and excluded participants throughout the study. ABI: ankle-brachial index, CCDS: color-coded duplex sonography, MSOT: multispectral optoacoustic tomography, HV: healthy volunteer, IC: intermittent claudication patient, CLTI: chronic limb-threatening ischemia patient, CKD: chronic kidney disease, PAD: peripheral artery disease, ROI: region of interest, US: ultrasound. Created with BioRender.com.

Between 11/13/2020 and 8/20/2021 a total of n = 239 participants were screened for inclusion, of whom n = 220 gave written consent and were enrolled in the study. The final study cohort (containing matched, complete data sets of both US/SWL-OAI and multispectral imaging data) consisted of n = 193 data sets. Parts of the multispectral dataset were reported before [30]. A total of n = 27 participants were excluded from study participation either already during screening examinations due to meeting clinical exclusion criteria or due to inappropriate measurement conditions (see Fig. 1).

**Fig. 2:**
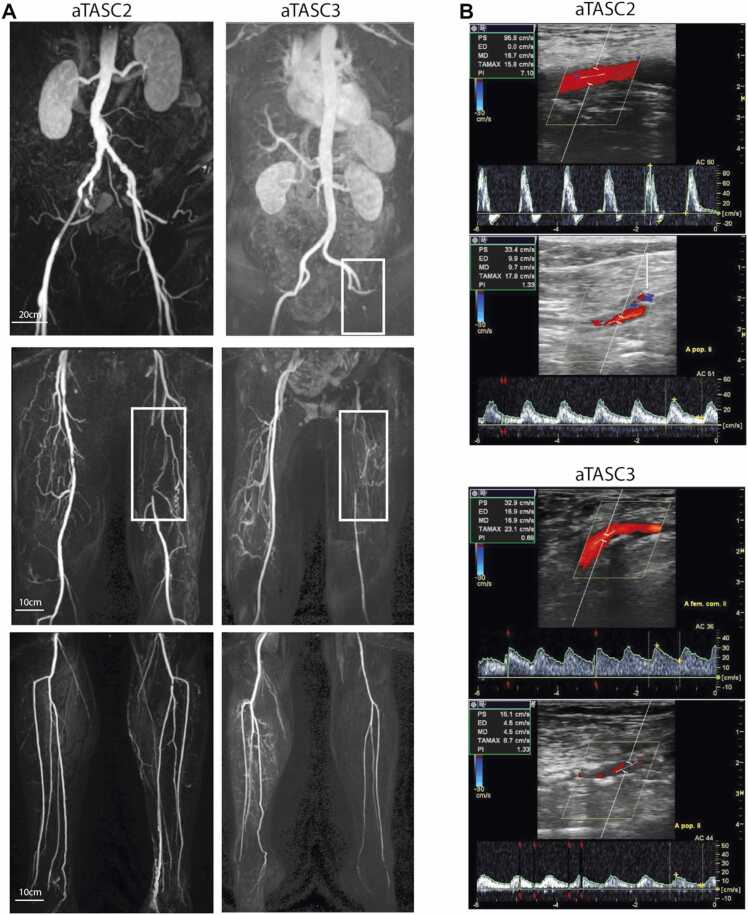
Representative imaging results. A Representative magnetic resonance angiography (MRA) images from an aTASC 2 (left) and aTASC 3 (right) patient. Top images: aorto-iliac (AI) vessel section, middle images: femoro-popliteal (FP) vessel sections and lower images: infrapopliteal (IP) vessel sections. White boxes outline detected stenoses. **B** Representative color-coded duplex sonography (CCDS) images from an aTASC 2 (top images) and aTASC 3 (bottom images) patient. The top image shows the flow profile and the measured peak systolic velocity (PSV) of the common femoral artery (CFA), respectively, and the bottom image of the popliteal artery (PA). The aTASC 2 images show a biphasic flow profile in the CFA and a monophasic flow profile in the PA, and the aTASC 3 images show a monophasic flow profile in the CFA and PA.

The mean age in the study cohort was 67.6 ± 10.3 years and 36.8% were women. Relevant demographic and clinical data of the study cohort is given in Table 1**.** For further analyses, the cohort was divided according to clinical severity (Fontaine classification, Table S1-3), resulting in n = 59 healthy volunteers (HV; no clinical PAD symptoms and normal ABI, palpable foot pulses), n = 76 intermittent claudication patients (IC; Fontaine IIa/IIb), and n = 58 patients with chronic limb-threatening ischemia (CLTI; Fontaine III/IV). Representative clinical standard imaging assessments for the PAD patient group in this study are demonstrated in Fig. 2.

**Table 1:** Baseline demographic and clinical data presented according to 3-level stratified clinical (Fontaine) classification and total study cohort.

Characteristic	Total sample n = 193	HV n = 59	IC n = 76	CLTI n = 58
**Age, yrs.**	67.6 ± 10.3	62.1 ± 9.3	67.5 ± 9.0	73.4 ± 9.9
**Female sex, n (%)**	71 (36.8)	30 (50.8)	24 (31.6)	17 (29.3)
**Risk factors for PAD, n (%)**				
**Smoking Elevated blood fat** **Obesity** **Positive family history**	75 (38.9) 92 (47.7) 26 (13.5) 69 (35.8)	4 (6.8) 12 (20.3) 3 (5.1) 25 (42.4)	44 (57.9) 46 (60.5) 15 (19.7) 30 (39.5)	27 (46.6) 34 (58.6) 8 (13.8) 14 (24.1)
**Relevant underlying diseases, n (%)**				
**Arterial hypertension** **Diabetes mellitus** **Coronary artery disease** **Carotid stenosis** **Cerebral insults**	145 (75.1) 40 (20.7) 51 (26.4) 21 (10.9) 15 (7.48)	29 (49.2) 0 (0) 4 (6.8) 0 (0) 4 (6.8)	61 (80.3) 16 (21.1) 19 (25.0) 11 (14.5) 5 (6.6)	55 (94.8) 24 (41.4) 28 (48.3) 10 (17.2) 6 (10.3)
**Previous vascular intervention, n (%)**	49 (25.4)	0 (0)	31 (40.8)	18 (31.0)
**Current medication, n (%)**				
**Lipid-lowering agent** **Antihypertensive** **Antidiabetic** **ASA**	85 (44.0) 138 (71.5) 33 (17.1) 88 (45.6)	10 (16.9) 27 (45.8) 0 (0) 9 (15.3)	44 (57.9) 58 (76.3) 15 (19.7) 52 (68.4)	31 (53.4) 53 (91.4) 18 (31.0) 27 (46.6)
**PAD stage according to Fontaine, n (%)**				
**HV** **IIa** **IIb** **III** **IV**	59 (30.6) 20 (10.4) 56 (29.0) 13 (6.7) 45 (23.3)	59 (100) 0 (0) 0 (0) 0 (0) 0 (0)	0 (0) 20 (26.3) 56 (73.7) 0 (0) 0 (0)	0 (0) 0 (0) 0 (0) 13 (22.4) 45 (77.6)
**PAD stage according to aTASC,** n (%)				
**aTASC 1** **aTASC 2** **aTASC 3**	59 (30.6) 116 (60.1) 18 (9.3)	59 (100) 0 (0) 0 (0)	0 (0) 71 (93.4) 5 (6.6)	0 (0) 45 (76.3) 13 (22.0)

HV: healthy volunteer (no previously known PAD or PAD-typical symptoms, ABI 1.0–1.3), IC: intermittent claudication (Fontaine stage IIa: claudication at a distance >200 m and IIb: claudication at a distance <200 m), CLTI: chronic limb threatening ischemia (Fontaine III: ischemic rest pain, mostly in the feet and IV: necrosis and/or gangrene of the limb), ABI: ankle-brachial index; ASA: acetylsalicylic acid; aTASC: aggregated Trans-Atlantic Inter Society Consensus score (aTASC 1: included as HV with normal clinical findings or no arteriosclerotic findings in angiography; aTASC 2: assumption of sufficient collateralization due to mild findings in aortoiliac or femoropopliteal section; aTASC 3: assumption of poor collateralization due to severe aortoiliac findings). Continuous factors are given as means ± standard deviation (SD), categorial factors in absolute and relative frequencies.

### 2.2 Comparison of SWL-OAI and multispectral OAI

**Fig. 3:**
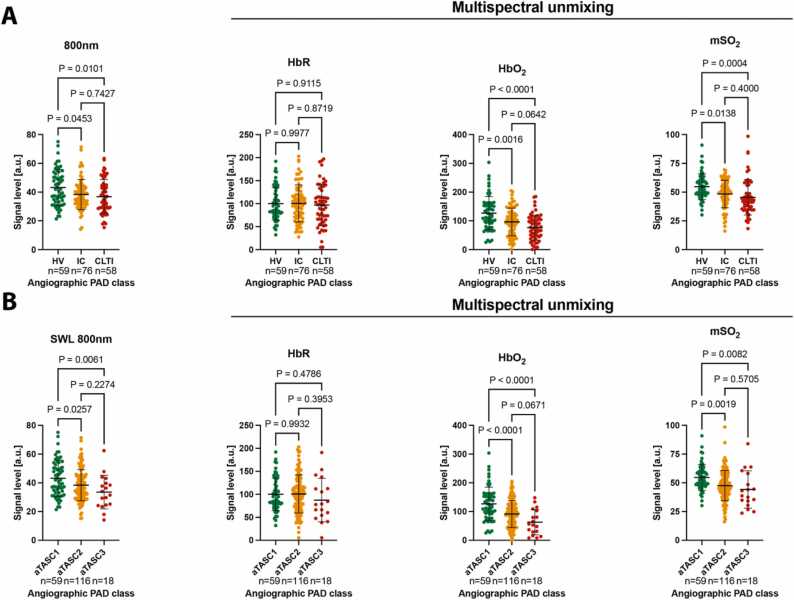
Single wavelength optoacoustic imaging at 800 nm compared to multispectral unmixed parameters. A Distribution of the acquired MSOT readouts for SWL 800 nm according to clinical severity of PAD (HV; no clinical PAD symptoms, normal ankle-brachial-index (ABI) and palpable foot pulses; IC; combined Fontaine IIa: claudication at a distance > 200 m and IIb: claudication at a distance < 200 m; CLTI; combined Fontaine III: rest pain, mostly in the feet and IV: necrosis and/or gangrene of the limb) compared to deoxygenated hemoglobin (HbR), oxygenated hemoglobin (HbO_2_), and multispectral unmixing derived oxygenation (mSO_2_). **B** Distribution of the acquired MSOT readouts for SWL 800 nm according to the angiographic severity (aTASC; aTASC1: included as healthy volunteer (HV) with no arteriosclerotic findings in angiography, aTASC 2: assumption of collateralization due to mild findings in aortoiliac (AI) or femoropopliteal (FP) vessel section, aTASC 3: assumption of poor collateralization due to severe AI findings) compared to deoxygenated hemoglobin (HbR), oxygenated hemoglobin (HbO_2_), and multispectral unmixing derived oxygenation (mSO_2_). Dots indicate individual datapoints from patients, error bars indicate mean±SD.

Frist, we investigated whether SWL-OAI was capable to differentiate the 3-level stratified clinical classification of PAD (Healthy volunteers (HV), intermitted claudication (IC), chronic limb-threating ischemia (CLTI)). Such a classification best reflects the classification of patients in routine clinical practice. This was compared to multispectral unmixed parameters deoxygenated hemoglobin (HbR), oxygenated hemoglobin and multispectral-derived oxygenation (msO_2_). We found SWL 800 nm was able to separate IC (38.3 ± 10.5 arbitrary units [a.u.], P = 0.0453) and CLTI patients (36.9 ± 11.8 a.u., P = 0.0101) from a higher signal in HV (43.2 ± 12.6). There was no difference within the two PAD groups. Similarly, HbO_2_ and mSO_2_ were capable to differentiate IC (P = 0.0016, P = 0.0138) and CLTI patients (P < 0.0001, P = 0.0004). In contrast, HbR did not show any significant differences between groups (Fig. 3A). These results were confirmed when using the aggregated angiographic disease severity as stratified by an aggregated classification derived from the Trans-Atlantic Inter-Society Consensus Document on Management of Peripheral Arterial Disease (TASC II) (aTASC). It reflects an inter-society consensus for the management of PAD and describes, among other things, the subdivision of the anatomic distribution of lesions on angiographic imaging (compare Supplementary Appendix Table S4). SWL 800 nm signal show a decrease from aTASC1 (43.2 ± 12.6 a.u.) to aTASC2 (38.4 ± 10.9 a.u., P = 0.0257) and aTASC3 (33.5 ± 11.7 a.u., P = 0.0061). As compared to clinical classification, there was a similar signal decrease with increasing angiographic disease severity in HbO_2_ and mSO_2_, while HbR remained unchanged (Fig. 3B). This shows the strong influence of oxygen-dependent parameters associated with chronic reduced blood flow in the leg.

### 2.3 Ultrasound grayscale levels for assessment clinical and angiographic PAD severity

**Fig. 4:**
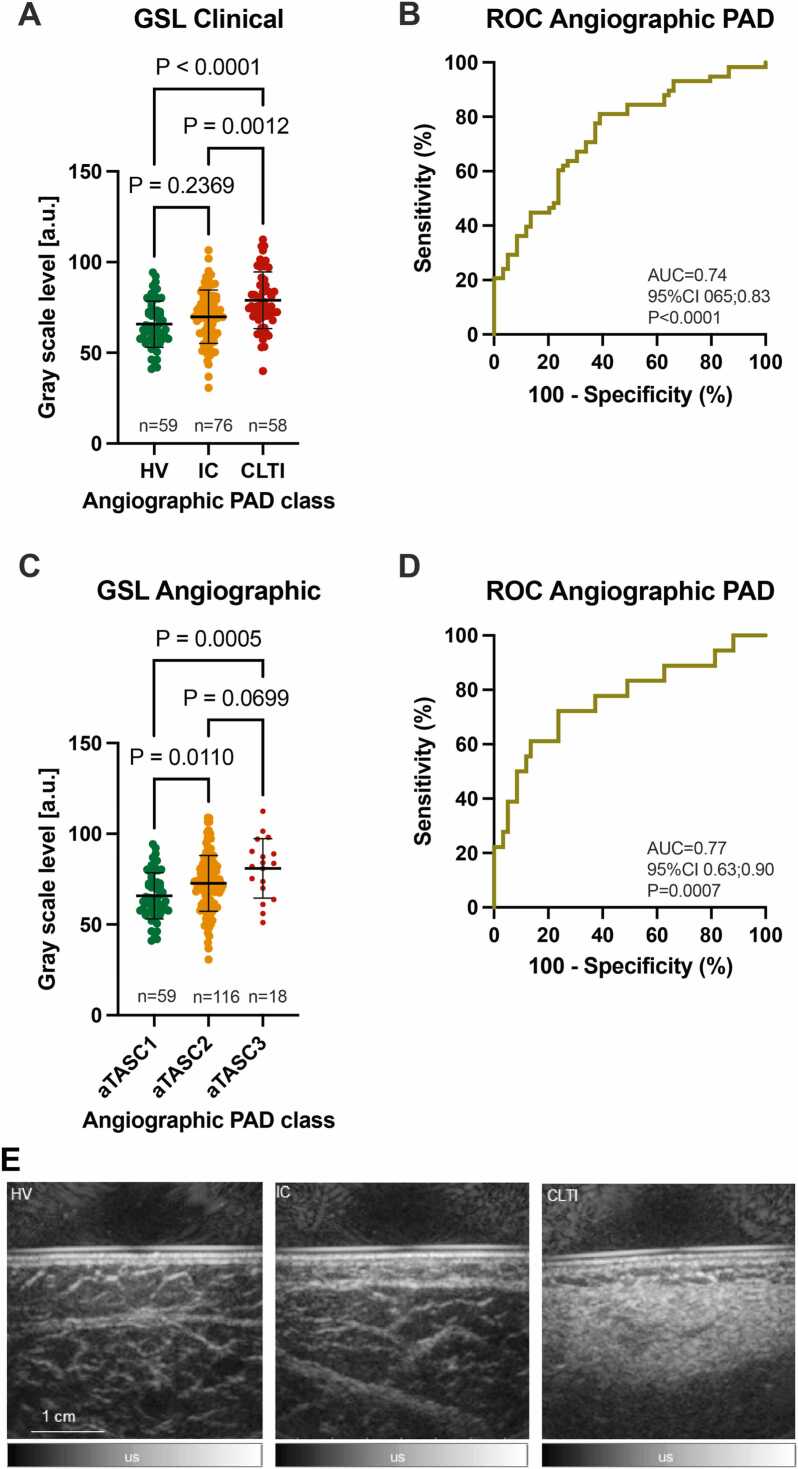
Quantitative ultrasound (US) assessment. A Distribution of gray scale pixel levels (GSL) by clinical severity of PAD (HV; no clinical PAD symptoms, normal ankle-brachial-index (ABI) and palpable foot pulses; IC; combined Fontaine IIa: claudication at a distance > 200 m and IIb: claudication at a distance < 200 m; CLTI; combined Fontaine III: rest pain, mostly in the feet and IV: necrosis and/or gangrene of the limb). **B** Receiver operating characteristic (ROC) curve with area under curve (AUC) and 95% confidence interval (CI) to distinguish between HV and CLTI for n = 193 subjects (n = 59 HV/ n = 58 CLTI). **C** Distribution of GSL according to angiographic disease severity (aTASC; aTASC1: included as healthy volunteer (HV) with no arteriosclerotic findings in angiography, aTASC 2: assumption of collateralization due to mild findings in aortoiliac (AI) or femoropopliteal (FP) vessel section, aTASC 3: assumption of poor collateralization due to severe AI findings). **D** ROC with AUC and 95% CI to distinguish between aTASC 1 and aTASC for n = 193 subjects (n = 59 aTASC1/ n = 18 aTASC3) using GSL analysis. **E** Exemplary ultrasound images of calf muscles of a HV (left image), an IC patient (middle image) and a CLTI patient (right image). A distinct increase in intensity of the ultrasound signal between the image of the HV and the CLTI patient can be observed.

As different stages of PAD are associated with reduction in walking capacity and physical function, we wanted to examine whether US grayscale levels (GSL) would be sufficient to differentiate clinical PAD severity. The distribution of US GSL readouts showed a significant increase from HV to CLTI patients (65.8 ± 12.8 arbitrary units [a.u.] vs. 78.9 ± 15.6 a.u., <0.0001). Similarly, there was an increase in mean US GSL between IC and CLTI patients (69.9 ± 14.7 a.u. vs. 78.9 ± 15.6 a.u., P = 0.0012) (Fig. 4A). However, no difference was found in HV and IC patients (P = 0.2369). The receiver operating characteristic (ROC) analysis showed an area under curve (AUC) of 0.74 (95%CI 0.65–0.83, P < 0.0001) for the distinction between HV and CLTI patients (Fig. 4B). Using the maximized Youden index, a cut-off of 67.9 a.u. was determined to differentiate the groups with a sensitivity of 81.0% and a specificity of 61.0%.

Next, aTASC classification was used to assess the distribution of US GSL readouts according to angiographic severity. With increasing aTASC level US GSL mean values showed an increase from aTASC1 (65.8 ± 12.8 a.u.) to aTASC2 (72.7 ± 15.4 a.u., P = 0.0110) and aTASC3 (81.0 ± 16.4 a.u., P = 0.0005), respectively (Fig. 4**C**). The ROC analysis resulted in an AUC of 0.77 (95%CI 0.63–0.90, P = 0.0007) for the distinction between HV and CLTI patients (Fig. 4**D**). A cut-off of 80.6 a.u. provided a sensitivity of 61.1% and a specificity of 86.4%.

### 2.4 Hybrid SWL-OAI for assessment of clinical and angiographic PAD severity

**Fig. 5:**
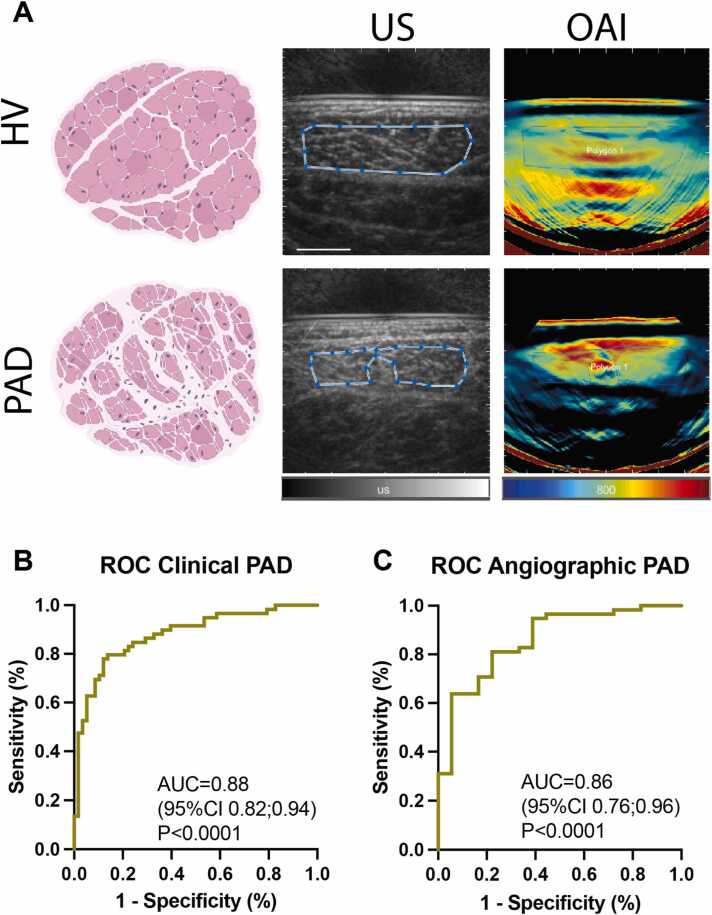
Hybrid US/SWL-OAI imaging. A Representative US and MSOT images from a healthy volunteer (HV; no clinical PAD symptoms and normal ABI) (top row) and a chronic limb threatening ischemia patient (CLTI; Fontaine III: rest pain, mostly in the feet and IV: necrosis and/or gangrene of the limb) (bottom row). Pink Illustration: sketch of a healthy (top) and fibrotic (bottom) skeletal muscle. Blue boxes show the polygonal region of interest (ROI) within the muscle tissue. Created with BioRender.com. **B** Receiver operating characteristic (ROC) curve with area under curve (AUC) and 95% confidence interval (CI) to distinguish between HV and CLTI for n = 193 subjects ((n = 59 HV/ n = 58 CLTI) using the combination of hybrid US/OAI, age, depth and gender. **C** ROC with AUC and 95% CI to differentiate aTASC 1 from aTASC 3 for n = 193 subjects (n = 59 aTASC1/ n = 18 aTASC3) using the combination of hybrid US/OAI, age, depth and gender.

Since US GSL showed an increase and SWL 800 nm a decrease in disease severity, we wanted to investigate if this two complementary signal information could be used in a hybrid approach (Fig. 5A). In comparison to multispectral unmixed parameters, this may reduce the clinical effort by acquiring less complex datasets. To assess its possible diagnostic accuracy, a multiple logistic regression was performed using US GSL, 800 nm OAI signals, the individual gender, age and imaging depth as explanatory factors. The maximum ROC (by including GSL+800 nm+gender+age+depth) resulted in an AUC of 0.88 (95%CI 0.82–0.94, P < 0.0001) to distinguish HV from CLTI (Fig. 5B). A full analysis for all factors can be found in Table S5. To differentiate aTASC1 from aTASC3 (by including GSL+800 nm+gender+age+depth), this approach resulted in a maximum AUC of 0.86 [95%CI 0.76–0.96] (P < 0.0001) (Fig. 5**C**). A full analysis for all factors can be found in Table S6.

### 2.5 Calf muscle biopsy sampling confirms muscle degeneration

**Fig. 6:**
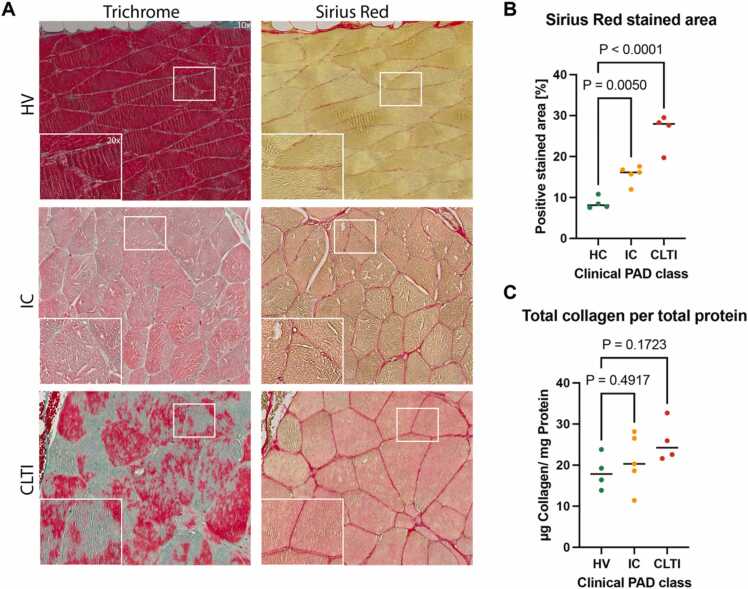
Calf muscle biopsy sampling confirms muscle degeneration in PAD. A Trichrome (TriC) and sirius red (SiR) staining results. Grouping was based on the clinical disease severity (HV; no clinical PAD symptoms, normal ankle-brachial-index (ABI) and palpable foot pulses; IC; combined Fontaine IIa: claudication at a distance > 200 m and IIb: claudication at a distance < 200 m; CLTI; combined Fontaine III: rest pain, mostly in the feet and IV: necrosis and/or gangrene of the limb). Musculature of the CLTI patient shows a more inhomogeneous pattern of myofibers of varying size and shape and increased collagen content (red/ turquoise). **B** Quantitation of positively stained area in SiR staining based on clinical disease severity. **C** Quantitation of total collagen per total protein derived by photometric assay. Independent samples t-test was used for statistical analysis. If normal distribution could not be assumed, the Mann-Whitney U-test was applied.

Changes in the US echogenicity and the intensity of the OA signals in muscles suggest that morphological changes in the muscle may occur in the context of PAD. To prove that muscle degeneration was also present in PAD patients, we assessed the degree of muscle degeneration by Trichrome and Sirius-Red (SiR) staining of muscle biopsies (Fig. 6A). These techniques enable the assessment of tissue remodeling by the measuring tehe area covered by extracellular matrix components. The proportion of positively stained area showed significant increase from HV (8.7 ± 1.5%) compared IC (15.7 ± 2.2%, P = 0.0050) and CLTI patients (26.3 ± 4.5%, P < 0.0001), respectively (Fig. 6B). The quantitative relative collagen content assessed by photometric measurement of hydroxyproline revealed an increase of collagen per total protein in IC (21.00 ± 6.7 µg/mg, P = 0.4917) and CLTI patients (25.7 ± 5.0 µg/mg, P = 0.1723), respectively, compared to healthy muscle samples (18.3 ± 4.3 µg/mg) (Fig. 6C).

### 2.6 Exercise-dependence of multispectral and SWL-OAI

Now the question remained open whether the considered measurements would change depending on a changed oxygen concentration. For this purpose, we analyzed again both SWL and multispectral data obtained after the patients were challenged by a defined walking distance. We hypothesized, that this would lead to a functionally measurable change in the oxygen-associated parameters, while the morphological 800 nm information would remain unchanged.

**Fig. 7:**
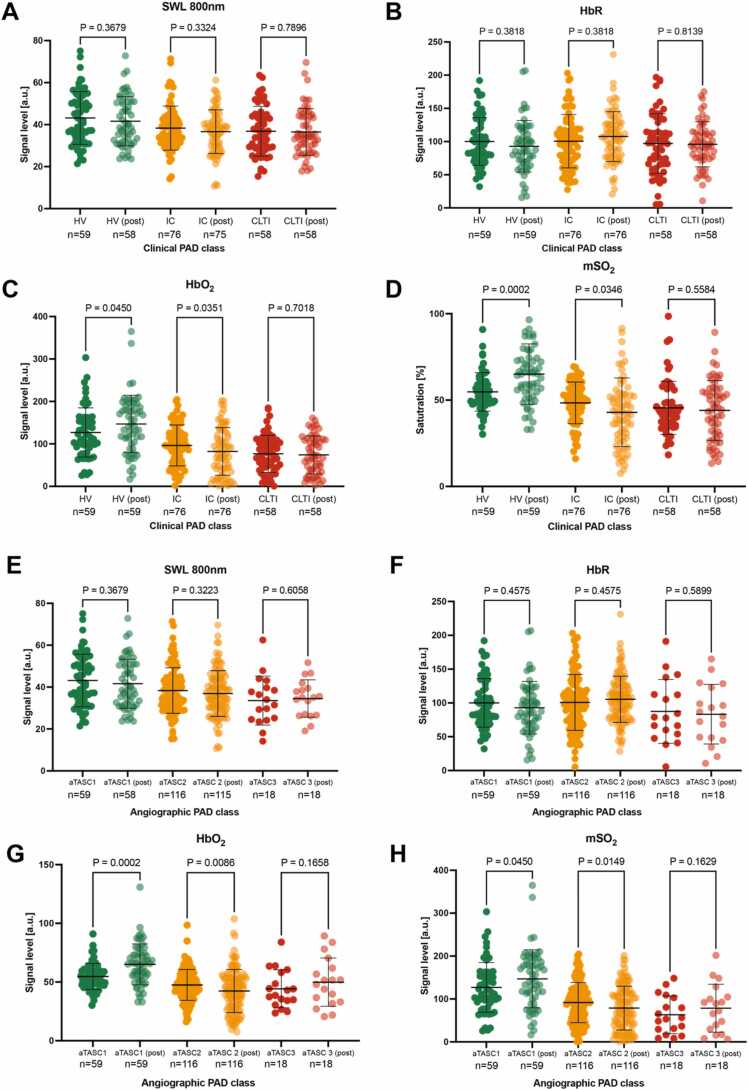
Exercise induced changes to optoacoustic signals. A-D Distribution of quantitative SWL 800 nm and spectral unmixed deoxygenated hemoglobin (HbR), oxygenated hemoglobin (HbO_2_) and multispectral unmixing derived oxygenation (mSO_2_) signals before and after exercise. The patients were grouped based on the clinical disease severity (HV; no clinical PAD symptoms, normal ankle-brachial-index (ABI) and palpable foot pulses; IC; combined Fontaine IIa: claudication at a distance > 200 m and IIb: claudication at a distance < 200 m; CLTI; combined Fontaine III: rest pain, mostly in the feet and IV: necrosis and/or gangrene of the limb). **E-H** Distribution of quantitative SWL 800 nm and spectral unmixed deoxygenated hemoglobin (HbR), oxygenated hemoglobin (HbO_2_) and multispectral unmixing derived oxygenation (mSO_2_) signals before and after exercise. The patients were grouped based on the angiographic disease severity (aTASC; aTASC1: included as healthy volunteer (HV) with no arteriosclerotic findings in angiography, aTASC 2: assumption of collateralization due to mild findings in aortoiliac (AI) or femoropopliteal (FP) vessel section, aTASC 3: assumption of poor collateralization due to severe AI findings). Dots indicate individual datapoints from patients, error bars indicate mean±SD.

Using the clinical PAD classes, we were able to measure an unchanged SWL 800 nm signal before and after performing the challenge across all severity stages (Fig. 7A). This was expected, since a SWL contains the morphological information. HbR showed similar outcome (Fig. 7B). In contrast, HbO2 and mSO2 both increased in HV (P = 0.0450, P = 0.0002) and decreased in IC (P = 0.0351, P = 0.0346) (Fig. 7C+D). Under angiographic classification, we could make similar observations within the three groups of patients (Fig. 7E-H). This most likely indicates a functional deficit of the muscle or an impaired vascular supply.

### 2.7 Adverse events

No adverse events have occurred throughout this study.

## 3 Discussion

As early as 1980, Heckmatt et al. described an increase in muscle ultrasound echogenicity as an imaging correlate of muscle tissue remodeling and fibrosis [33]. In this study, we aimed to visualize structural muscular changes in calf muscles of PAD patients noninvasively using a hybrid ultrasound/OAI approach. Imaging results correlated significantly with clinical and angiographic disease severity, as well as to the recently published unmixed multispectral data [30]. These imaging findings were verified by histologic and photometric analyses of muscle biopsies, revealing an increase in tissue remodeling, reflecting degeneration of the calf muscle according to PAD severity.

According to our data, standardized ultrasound images from the hybrid imaging system can be used to distinguish HV from CLTI patients. Similar ultrasound findings were seen by Zaidman et al. in severe inherited muscular diseases [21], suggesting also a relevant muscle pathology in PAD. As ultrasound imaging is a clinical standard in PAD diagnostics [34], this is an ideal basis to apply these noninvasive and quantitative imaging techniques to objectify long-term outcome measures. Compared to clinical US, the integrated RUCT in this device does not allow to change imaging parameters (gain, depth compensation, field of view) resulting in standardized US imaging data [35].

Unmixed multispectral data of OAI in PAD diagnostics is currently used to measure hemoglobin parameters as indicators of muscular microcirculation [24]. We investigated the potential of a SWL-OAI approach to detect muscle degeneration in PAD patients. Replacement of healthy tissue and subsequent fibrosis have been shown to occur in PAD [5], [7], [14], [36], [37]. In this study, we used a simple approach with a single wavelength of 800 nm to image the degeneration of calf muscles [32]. As shown by our phantom experiment, this does not only provide an assessment of the total hemoglobin signal at the isosbestic point [38], [39] but also the myoglobin content in the investigated area. Therefore, SWL-OAI signals may contain information about overall viable muscle tissue and the amount of present blood. This assumption coincides with study by Lin et al. finding a high proportion of optical absorption in muscle tissue is caused my myoglobin [40].

A major advantage of this cohort is, that we were able to compared SWL-OAI to meaningful multispectral unmixed data that has already been investigated [30]. Beyond the multispectral unmixed parameter of oxygenated hemoglobin, we have now added the parameters HbR and mSO_2_. Interestingly, SWL-OAI is already sufficient to separate the clinical and angiographic disease groups. The addition GSL imaging data further increases the diagnostic discriminatory accuracy. While, as hypothesized, SWL-OAI only tends to map tissue morphology, multispectral parameters can also derive functional changes in muscle tissue. Accordingly, the chronic changes caused by chronic limb ischemia led to a more pronounced decrease in oxygen saturation during exercise. Nevertheless, these functional changes were especially found in the group of less affected patients. This could open a window for early noninvasive diagnostics, which may be captured because the degeneration of the muscle is not yet far advanced. Another explanation could be that patients with a higher stage of the disease are hardly able to correctly exercise.

Furthermore, the SWL 800 nm was found to be less prone to alteration by penetration depth as compared to a direct multispectral imaging approach to derive collagen signals [22]. Limited penetration depth due to variations in light absorption and scattering might also be more prominent in obese patients, patients with skin abnormalities, and infection or edema of the targeted region. As seen in our images, this impacts the uniformity of the optoacoustic signal.

In a clinical context, it may become important, which diagnostic test is performed first. A serial procedure (first US, then OAI) could be used to avoid one of the procedures (in this regard OAI). In the age of the hybrid US/OA systems, this is not necessarily due to the simplicity and non-invasiveness of the approach. Therefore, parallel testing (simultaneous US/OA) could be used to either increase sensitivity or specificity by defining the dependence of one on the other test.

Currently, main outcome parameters in the treatment of PAD patients are amputation free survival (AFS), major adverse limb events (MALE) in the long term follow up as well as the ankle or toe brachial index results, assessed directly after revascularization. Additional, non-invasive diagnostic methods like transcutaneous oxygen partial pressure (TcPo_2_) or indocyanine green fluorescence angiography focus on the skin perfusion of the affected limb, mainly investigating dynamic and therefore physiological changes accompanying PAD [14], [15], [37], [41]. However, neither of these measures directly represent the end organ damage in calf musculature nor can they be applied to all patients, nor can they reflect non-invasively chronic muscle changes. The approach to identify end organ damage directly might allow a much more specific assessment and could potentially identify patients at high risk for critical disease progression or limb loss at earlier points.

This study has several limitations. Due to physiology and muscle pathology the obtained US GSL ROIs vary in depth and size, which may affect the measured GSL values [18]. To improve the measures of correlation to direct muscular damage, there should be direct comparison to MRI muscle imaging in future studies. HV did not receive angiographic imaging due to ethical concerns, so PAD had to be ruled out based solely on noninvasive diagnostic results. ABI measurements could not be completed in all patients due to pain or open wounds. Not all participants were able to complete the treadmill examination due to a variety of walking restrictions, which impedes the comparability of the results. Ischemic walking pain and ulcers may have led to restricted blinding of the examiners. The number of participants who underwent muscular biopsy was low due to the exploratory character of the study and may not be entirely representative of the whole sample of PAD patients. The possible influence of different skin types could not be investigated, because in the study population consisted exclusively of light skin types. The influence of different skin melanin content warrants further investigation for improved standardization [42].

In summary, this study demonstrated the feasibility of quantitative US/SWL-OAI to measure chronic muscle tissue degeneration as a consequence of chronic mal-perfusion. The work shows that SWL signals are most likely to map muscle degeneration, while multispectral measurements can be used to derive functional oxygen-dependent parameters.

## 4 Methods

### 4.1 Trial design

This prospective, monocentric diagnostic cross-sectional study was conducted at the Department of Vascular Surgery of the University Hospital Erlangen, Germany, between 11/13/2020 and 08/20/2021 (Fig. 1). Study approval was granted by the local ethics committee of the University Erlangen-Nürnberg (354_20 B and 285_21 B). The study adhered to the STARD guidelines [43]. The study was registered at clinicaltrials.org (ID NCT04641091) as reported in a separated approach [30]. All investigations were performed at a single visit per participant. There were included HV and PAD patients. Inclusion criteria in the PAD group involved: symptomatic PAD stages (Fontaine II-IV) and angiographically confirmed arteriosclerosis. The hemodynamic relevance of the stenosis was confirmed by additional color-coded duplex sonography (CCDS). The stenosis was defined as hemodynamically relevant above 50%, leading to a post-stenotic flow profile in the CCDS. In the HV group, normal ankle-brachial index (ABI) results (ABI 1.0–1.3), palpable foot pulses, no previously known symptoms typical of PAD, and no diabetes mellitus or chronic renal insufficiency were required.

For histologic comparison, in n = 9 patients muscle biopsy specimens were taken during surgery. Additionally, 4 healthy control samples were used for comparison. For more detailed information see Supplementary Appendix.

### 4.2 Clinical assessment

Relevant medical history and the vascular status were obtained by noninvasive diagnostics according to international guideline recommendations [34]. This included a vascular CCDS, ankle-brachial index (ABI, Table S7), pulse status and treadmill examination (see Supplementary Appendix).

### 4.3 Angiographic assessment

The latest angiographic images available of all PAD patients were reviewed by two independent investigators (A.T., J.G.) based on the Inter-Society Consensus for the Management of Peripheral Arterial Disease (TASC II) [44]. This could be either Magnetic resonance angiography (MRA), Computed tomography angiography (CTA) or Digital subtraction angiography (DSA) imaging. In case of discrepancies, a third experienced investigator (U.R.) was consulted for final decision. HV did not receive angiographic imaging. Aortoiliac (AI), femoropopliteal (FP) and infrapopliteal (IP) vessel sections were each scored according to TASC II. As this classification considers the respective vessel sections separately, the findings were combined, resulting in three aggregated TASC (aTASC) subgroups: aTASC 1: angiographically healthy, aTASC 2: collateralization capability, aTASC 3: poor collateralization capability (see Table S6).

### 4.4 Quantitative US assessment

Comparable to standard B-mode imaging, muscle US was performed using the integrated Reflected Ultrasound Computed Tomography (RUCT) of the US/OAI system (MSOT Acuity Echo, iThera Medical GmbH, Munich, Germany) [45]. For image analyses, the medial gastrocnemius muscle was visualized in a transversal imaging plane. To investigate muscle echo intensity (EI), RUCT images were analyzed by two independent investigators (A.T., J.G.) and reviewed by a third investigator (U.R.), each blinded towards other test results. A polygonal region of interest (ROI) was drawn directly beneath the muscle fascia excluding visible blood vessels and gray scale pixel levels (GSL) were derived by FIJI medical imaging software [35], [46]. Black and white pixels corresponded to values between 0 and 255 arbitrary units (a. u.) and standard mean GSL values were obtained for further analyses. In PAD patients, either the affected or more severely affected leg was chosen as targeted leg for analysis. In HV, a randomly selected leg was defined as target leg.

### 4.5 MSOT data acquisition

All MSOT data were generated with the identical US/OAI device (MSOT Acuity Echo, iThera Medical GmbH, Munich, Germany). The system was equipped with a handheld probe providing a center frequency of 4 MHz, field of view of 40mmx40mm (lateral x depth), a resolution for depth of 10 mm ( ± 5 mm) in the lateral image center ( ± 2 mm), < 1 mm in-plane (xy) and 2.5 mm out-of-plane (z). The inter-element pitch of the 2D handheld probe was 0.34 mm (256 elements and angular coverage of 125°) [47]. The participants were transferred to the examination room by wheelchair to avoid physical activity before the measurement. For imaging, they were placed in prone position. Before imaging, all hair was gently removed. The handheld probe was placed on the skin surface of the upper third of the calf in a transversal plane and the medial head of the gastrocnemius muscle was identified [22], [48]. Coupling was ensured by using transparent ultrasound gel (Aquasonic CLEAR, Parker Laboratories, Inc., USA). The entire measurement was then performed over a duration of 20 s

Subsequently, the study participant was asked to walk a defined distance of 150 m (incline 0%) without interruption to provoke increased muscle perfusion. A research assistant walked with the participant to provide support if needed and to set the best possible pace for the participant. For study participants who could not walk 150 m between both MSOT measurements the walking load was reduced in a staggered manner (reduced to 100, 50, or 25 m or, if the patient was completely unable to stand or walk: over two minutes extensions and flexions of the feet in a seated position under the guidance of the examiner). Immediately after the provocation test the MSOT examination was repeated as described above.

### 4.6 MSOT data analysis

MSOT imaging data comprised signals from multiple wavelengths ranging from 680 nm to 1100 nm (680, 715, 730, 760, 800, 850, 930, 950, 980, 1000, 1030, 1064 and 1100 nm), which were reconstructed from raw optoacoustic data using the standard back projection algorithm [22]. A low-pass band-pass filter of 4 MHz and high-pass band-pass filter of 5 kHz was applied. The algorithm for the US reconstruction was different from the one used for optoacoustic signals [49]. For post-processing of images, we used a mixture of color maps, brightness and contrast adjustments, and transparency for allow visualization of multiple layers. All the images were processed the same way. The study cohort was examined under two different approaches. On the one hand, hybrid US/SWL-OAI data were obtained and, at the same time, multiple wavelengths were acquired to derive spectral unmixed parameters oxygenated hemoglobin (HbO_2_), deoxygenated hemoglobin (HbR), and MSOT-derived oxygenation (msO_2_). While US/SWL-OAI should be used to derive the morphological changes in the musculature, the multispectral information was used to describe disease-related changes of hemoglobin and oxygenation in the calf muscle. Parts of the multispectral dataset was recently published [30]. However, to facilitate easier clinical adaption, the aim of this study was to use US/SWL-OAI at 800 nm [30], which has been used in previous studies to visualize disease-dependent tissue changes [32]. For better comparability, a parallel comparison of both approaches was carried out throughout. Analyses were performed from two displayable frames with well captured muscle and few motion artifacts by A.T. and J.G. and reviewed by two independent investigators (U.R., F.K.) using cLabs software (V2.67, iThera Medical GmbH). As described before [22], [23], [32], [48], [50], the reconstruction was performed from raw data using a standard back-projection algorithm, band-pass filtering and deconvolution with the electrical impulse response of the imaging probe. For compensation of light attenuation and improvement of visualization of deeper structures, light fluence was modelled using exponential decay using light scattering and absorption coefficients of μ_s_ = 10 cm^−1^ and μ_a_= 0.022 cm-^1^ at an average oxygen saturation of 70% [51]. For single wavelength imaging the oxygenation saturation is not necessary or used. Based on the ROI for the GSL analysis of the US-image, a comparable polygonal ROI was drawn directly underneath the muscle fascia excluding macroscopically visible blood vessels. Quantification was performed by iLabs software (iThera Medial GmbH, Germany) in a semi-automated batch-processing mode. Within the ROI, the MSOT signals corresponded to the mean signal value and were expressed in arbitrary units (see Supplementary Appendix).

### 4.7 Histologic assessment

Muscle tissue sampling was performed intraoperatively in n = 9 patients after obtaining written informed consent. Additionally, n = 4 healthy muscle tissue samples were randomly provided by the Department of Pathology of the University Hospital Erlangen. A sample of the medial gastrocnemius muscle was taken after all necessary surgical steps were performed, but before the surgical field was closed. Samples were divided in half and transferred into a vessel containing 4% formaldehyde solution in phosphate-buffered saline and into a container for cryopreservation. Hematoxylin-eosin (HE) stain, Sirius red stain (SiR) and Masson trichrome stain (TriC) were performed for histological collagen quantification. Positively stained tissue was calculated as fraction of the whole image. Each sample was also analyzed for total collagen and total protein content using a total collagen and total protein assay (both QuickZyme Biosciences, Netherlands) based on the quantitative colorimetric determination of hydroxyproline residues, obtained by acid hydrolysis of collagen [52]. The absolute collagen content was derived (after calibration) from the quotient between total collagen and total protein (see Supplementary Appendix).

### 4.8 Statistical analysis

Continuous variables are given as means with standard deviation. Categorical variables are given in absolute and relative frequencies. For correlations of continuous variables, Pearson’s correlation coefficient is used, while correlations with categorical factors are given by Spearman’s rho. In all statistical analyses, P < 0.05 is considered statistically significant. Prior to the analyses, data was tested for normal distribution using the Shapiro-Wilk test. Based on data collected until 03/05/2021, we estimated a sample size of 36 (wave length 800 nm) in each clinical group (80% power, alpha of 0.05 one-sided). The final MSOT data set was normalized using z-scores due to a one-time maintenance of the device by the manufacturer during data collection (05/12/2021). Group differences between the three merged clinical PAD classes as well as aTASC groups were tested for significance using the Kruskal-Wallis test. To minimize type I error, the Tukey correction was applied. Pre and post differences were assessed by pairwise comparisons using a mixed-model approach and similar post-hoc testing.

If statistically significant, a receiver operating characteristic (ROC) analysis was performed for the respective parameter to determine the area under the curve (AUC) and the accompanying 95% confidence interval (CI). Cut-off values are identified by determining the highest possible Youden index (YI), that measures the methods ability to balance sensitivity and specificity. This was calculated as follows: YI=Sensitivity+Specificity−1.

Independent sample t-test was used to determine significant differences between two groups. In cases of violated normal distribution the Mann-Whitney U-Test was applied. Single missing values were excluded from the respective sub analyses. If any muscle measurement of the target leg was unsuitable for the corresponding analysis, the entire subject was treated as a dropout. Multiple logistic regression was performed using GSL, SWL 800 nm, and the individual gender as independent, and clinical (HV vs CLTI) or angiographic (aTASC1 vs aTASC3) classification, respectively, as dependent variable. The individual gender was deemed as important independent variable as the optoacoustic muscle signal was reported to be significantly different between female and male subjects [32]. All statistical analyses were performed using IBM SPSS Statistics, version 28 (IBM Corp., N.Y., USA) or GraphPad Prism, version 9.4.0 (GraphPad Software, CA, USA).

## CRediT authorship contribution statement

M. J. W., F. K. and U. R. conceived the idea for the study. M. J. W., J. W., M. F. N., F. K., U. R. developed, wrote and submitted the trial protocol. A. P. T., A. S. G., W. L., A. M., J. K., M. C., Y. L., U. R. screened and recruited the participants. A. P. T., A. S. G. performed the imaging studies. R. T., D. K., M. U., M. J. W. provided technical support. M. E. provided histology samples. Y. H. performed histologic procedures and analyses. A. P. T., A. L. W., A. P. R., L. T., U. R., V. D., F. K. analyzed the data. A. P. T., F. K., U. R. and W. U. performed statistical analyses. F. K. and U. R. jointly supervised the work. All authors approved the final version of the manuscript.

## Declaration of Competing Interest

The authors declare the following financial interests/personal relationships which may be considered as potential competing interests: Knieling, Ferdinand reports a relationship with iThera Medical GmbH that includes: consulting or advisory and travel reimbursement. Maximilian J. Waldner reports a relationship with iThera Medical GmbH that includes: travel reimbursement. Adrian P. Regensburger reports a relationship with iThera Medical GmbH that includes: travel reimbursement. Ferdinand Knieling reports a relationship with Sanofi that includes: funding grants, speaking and lecture fees, and travel reimbursement. Adrian P. Regensburger reports a relationship with Sanofi that includes: speaking and lecture fees and travel reimbursement. Ferdinand Knieling has patent A device and a method for analyzing optoacoustic data, an optoacoustic system and a computer program issued to EP 19 163 304.9. Ferdinand Knieling is serving as a Section Editor for Photoacoustics.

## Data Availability

Data will be made available on request.
