# Supplementary material for: Hybrid ultrasound and single wavelength optoacoustic imaging reveals muscle degeneration in peripheral artery disease
Source: Photoacoustics. 2023 Dec 2;35:100579. doi: 10.1016/j.pacs.2023.100579 (PMC10835356; doi:10.1016/j.pacs.2023.100579)
Supplement: Supplementary file 1 — Supplementary material [file mmc1.docx]

**Supplementary appendix**

Supplement to: Träger A, et al. **Noninvasive Staging of Muscle Degeneration in Peripheral Artery Disease using Single Wavelength Hybrid Optoacoustic Imaging**

This appendix has been provided by the authors to give readers additional information about their work.

Table of Contents

Material and methods 3

**Study procedures** 4

*Quantitative muscle ultrasound* 4

*MSOT data acquisition* 4

*MSOT data analysis and image representation* 5

**Histological assessments** 6

*Muscle biopsy sampling* 6

*Histologic muscle fibrosis quantification* 6

*Collagen quantification through photometric hydroxyproline determination* 6

**Clinical assessments** 7

*Relevant medical history* 7

*Ankle-brachial-index measurement* 7

*Color coded duplex sonography* 7

*Treadmill examination* 8

*Clinical staging* 8

*Blinding* 8

**Statistical analysis** 9

*Handling of missing data* 9

Results 10

Supplementary Figures 11

Supplementary Tables 14

References 23

Material and methods

**Eligibility criteria**

Patients eligible for inclusion were adults (> 18 years), able to give their written consent, with manifest PAD stages IIa, IIb, III or IV according to Fontaine or category 1-6 according to Rutherford. Fontaine I and Rutherford 0 were excluded from study participation because they are asymptomatic stages that are not typically present in consultation hours. In all PAD patients, an angiography that had been previously performed as part of routine diagnostics (independent of the study), in accordance with current guidelines or had been indicated and the patient had given consent, was required. Ten additional patients with an indicated vascular surgery of the vascular segment below the knee as part of routine treatment were required for the purpose of muscle biopsy sampling with histological workup. Potentially eligible patients were identified through reviewing the ambulatory schedule of the Outpatient Clinic for Vascular Surgery of the University Hospital Erlangen as well as the Occupancy schedule of the Vascular surgery ward and then screened for inclusion through reviewing the according patient records. HV were recruited through the ambulatory consultation hours as well as the inpatient occupancy schedule if they were scheduled for a different vascular problem and PAD was not to be expected. HV were eligible if neither PAD, diabetes mellitus, chronic renal insufficiency nor symptoms in the sense of an intermittent claudication were previously known of. The ABI measurement had to claim a normal value and palpable foot pulses were required. In HV, the ABI was performed as a screening examination prior to study inclusion. For HV, angiography or an indicated surgery were not required. After the exclusion criteria were ruled out, potential candidates were informed about the possibility to participate in the study and if the disposition to participate was present, written consent was collected. All potential patients and HV were evaluated and screened for study entry by the study investigator during beforementioned office hours and based on their accessibility to the study investigators A.T. and J.G. (Monday to Friday between 8 pm and 5 pm). It was a convenient sample of patients with PAD and HV.

**Study procedures**

*Quantitative muscle ultrasound*

For ultrasound detection, 256 ultrasound transducers with a center frequency of 4 MHz (60% bandwidth), organized in a concave array of 125-degree angular coverage and a radius of curvature of 4 cm, were used. The MSOT-generated RUCT-images captured the medial head of the gastrocnemius muscle in a transversal plane. In PAD patients the affected or more affected leg was declared the target leg and in HV this decision was made randomly. RUCT-images of the target leg were selected from each participant and were then analyzed for gray scale pixel level (GSL) content using FIJI medical imaging software [1] to assess muscle echogenicity. A polygonal ROI was drawn in directly underneath the muscle fascia excluding macroscopically visible blood vessels. The analysis obtained measured area, minimum, maximum, and mean pixel values. For all further analyses, mean pixel values were chosen. Black and white pixels corresponded to values of 0 and 255 arbitrary units.

*MSOT data acquisition*

All MSOT images were obtained with one MSOT device (MSOT Acuity Imaging system; iThera Medical GmbH, Munich, Germany), located in the Medical Clinic 1 (Department for Gastroenterology) of the Internal Medicine Center of the University Hospital Erlangen. All participants were transported to the examination room by wheelchair to avoid physical activity of the calf muscle before the measurement. Patients and all examiners in the room were protected with laser safety goggles during the examination. Participants were asked to lie down in a prone position on a basic examination couch. If that was not possible due to e.g., immobility or pain, the examination could alternatively take place in a sitting position. Both legs were examined consecutively. To objectify the process and to avoid the susceptibility to errors, the first leg to be examined was always the right leg. The medial part of the gastrocnemius muscle was initially identified visually by the study investigator and then verified through the integrated RUCT image. For the MSOT acquisition, the probe was placed on the skin surface. To optimize the coupling of the transducer onto the body surface, existing body hair was shaved, and transparent ultrasound gel was applied. In case of skin abnormalities, such as hyperpigmentation, scars or nevi, these areas were specifically excluded from measurements. Once the region of interest (ROI) was identified, the position of the probe was marked by a skin pencil to ensure a stable scanning position and to allow measurement at the same site of the contralateral leg. The measurement was then performed over a duration of 20 seconds with verification of measurement stability by reviewing the integrated motion indicator. The contralateral leg was then measured identically. In PAD patients, the affected or more affected leg was declared as the target leg and the other leg as the nontarget leg, in healthy volunteers this categorization was made randomly. Measurements were then named on the device, including the study ID of the participant and the leg labeling.

*MSOT data analysis and image representation*

The measurement obtained MSOT signals at 680 nm, 715, 730, 760, 800, 850, 930, 950, 980, 1000, 1030, 1064 and 1100 nm, which were reconstructed from raw optoacoustic data using the standard back projection algorithm [2]. A.T. and J.G. performed the analysis using cLabs software (V2.67, iThera Medical GmbH). Two representable frames with well delineated muscle and few motion artifacts (motion indicator <0,75) were chosen for the analysis. A polygonal region of interest (ROI) based on the ROI for GSL analysis was drawn in directly underneath the muscle fascia of the medial gastrocnemius muscle according to the RUCT-image (see Figure S1). Visible blood vessels, the muscle fascia or other artifacts were explicitly excluded from the ROI. The mean value from both selected frames was then calculated. Quantification was performed by iLabs software (iThera Medial GmbH, Germany) in a semi-automated batch-processing mode. Within the ROI, the MSOT signals correspond to the mean signal value and are given in arbitrary units (a.u.). Multiple single wavelengths from the extended near infrared spectrum (680 nm, 715 nm, 730 nm, 760 nm, 800 nm, 850 nm, 920 nm, 1000 nm, and 1064 nm) were spectrally unmixed to determine the MSOT value collagen, representing a specific spectral signature from multispectral measurements. The cLabs analysis revealed six single wavelengths (800 nm, 930 nm, 1000 nm, 1030 nm, 1064 nm, 1100 nm) for each frame evaluated, as well as the spectrally unmixed parameters collagen and lipid.

**Histological assessments**

*Muscle biopsy sampling*

With the purpose of evaluating an independent PAD cohort for its intramuscular collagen content to determine the extent of muscle degeneration in PAD patients, 10 independent PAD patients were selected for an intraoperative muscle biopsy. A total of ten patients with an indicated surgery, within which an unobstructed access to the medial head of the gastrocnemius muscle was to be expected during surgery, were selected for muscle biopsy sampling. Provided that verbal and written consent were given, the collection of a muscle tissue sample was considered, and the leading surgeon was informed. The biopsy was taken after all necessary surgical steps were performed, but before the surgical field was closed and only if the surgical field gave unobstructed access to the medial head of the gastrocnemius muscle. Given all these conditions, the surgical tissue sampling could be performed. A muscle tissue sample of the medial gastrocnemius muscle, approximately 10 mm long and 5 mm in diameter, was taken and divided in half with a scalpel. The two samples were then transferred into a vessel containing 4% formaldehyde solution in phosphate-buffered saline and into a container for cryopreservation. The biopsies were pseudonymized using a four-digit code. In addition, three paraffin-embedded muscle tissue samples from healthy controls from the tissue bank of the pathological institute of the University Hospital Erlangen were likewise examined.

*Histologic muscle fibrosis quantification*

After histological tissue sections had been prepared from the muscle tissue samples, hematoxylin-eosin (HE) stain, Sirius red stain (SiR) and Masson trichrome stain (TriC) were performed. SiR and TriC stained sections were examined for collagen content as follows. The sections were imaged using a tenfold magnification microscope. The according TIF files were exported and analyzed using FIJI software (v.2.0.0 or later, available at hhtps://fiji.sc). Five fields of view of each sample were analyzed and subsequently averaged. The images were divided into three channels (red, blue, and green). For collagen quantification, the red and green channels were used for TriC and SiR staining. Positively stained tissue was calculated as a fraction of the whole image.

*Collagen quantification through photometric hydroxyproline determination*

Four paraffin-embedded muscle tissue sections (10 micrometers) from each sample were analyzed for their absolute collagen content. A total collagen assay (QuickZyme Biosciences, Netherlands), based on the quantitative colorimetric determination of hydroxyproline residues was used to infer the amount of collagen in the sample. The measured hydroxyproline residues represent all types of collagens that are present in the sample, including procollagen, mature collagen, and degradation products [3]. For the correct interpretation of the obtained data, they should be compared with a reference variable like total protein in the sample. Therefore, a total protein assay (Quickzyme Biosciences, Netherlands) that determines the total amount of amino acids in the hydrolysate, with exception of proline and hydroxyproline, was performed. According to the manufacturer’s instructions, the muscle tissue samples were acid hydrolyzed overnight in 6M hydrochloric acid at 95°C. Samples for total collagen and total protein content were measured using a microplate reader at 550 nm. The absolute collagen content was derived (after calibration) from the quotient between total collagen and total protein.

**Clinical assessment****s**

*Relevant medical history*

All study relevant medical history was collected through reviewing the according electronic patient files and performing a complementary anamnesis interview with the study participant. Queried were the vascular risk factors smoking, elevated blood fat, obesity, positive family history, the underlying diseases arterial hypertension, diabetes mellitus, coronary artery disease, heart failure, atrial fibrillation, preterminal and terminal chronic kidney disease (CKD), carotid stenosis, and cerebral insults. Previous performed vascular surgeries (open surgeries, interventional or both) and a selection of the currently taken medication (Lipid-lowering agents, Antihypertensives, Antidiabetics, Acetylsalicylic acid (ASA), Clopidogrel, Heparin, Oral anticoagulant, Coumarin, Naftidroforyl, Cilostazol, Prostanoid) were also queried.

*Ankle-brachial-index measurement*

After a resting period of at least 10 minutes in a supine position, the ancle-brachial index (ABI) measurement was performed in all patients and HV. Prior to the ABI measurement, a pulse status of both common femoral arteries (CFA), popliteal arteries (PA), dorsalis pedis arteries (DPA) and posterior tibial arteries (PTA) was obtained and documented as “present” or “absent”. The occlusion pressure in mmHg of both brachial arteries, DPA and PTA was determined with a handheld cw-doppler using a standard blood pressure cuff. The ABI value was calculated based on the recommendations of the national S3 guideline [4] and the international ESC Guidelines in collaboration with the European Society for Vascular Surgery (ESVS)[5].

*Color coded duplex sonography*

Color-coded duplex sonography (CCDS) of both common femoral and popliteal arteries was performed in all participants by A.T. or J.G. using dedicated high-end ultrasound devices (Logic P6 Pro; GE Healthcare; Linear probe 9L, 9 MHz and Logic E9; GE Healthcare; Linear probe 9L-D, 9 MHz). For this examination the participants were asked to lie down in a supine position on a basic examination couch. Always starting on the right side, both common femoral arteries (CFA) were examined first. Both popliteal arteries (PA) were then examined in the same order. Standard ultrasound gel was applied for optimal coupling of the transducer First, the respective vessel was located in the B-mode and adjusted in cross-section. Then the color mode was activated, and the vessel was displayed in longitudinal section so that it was set diagonally in the image if possible. The peak systolic velocity (PSV [cm/s]) and the arterial flow profile (graded as monophasic, biphasic, triphasic or suspicion of occlusion) were recorded and documented. Since CCDS is considered the diagnostic method of first choice to clarify the aorta and its branches[4] as well as the iliac and leg arteries, it was determined whether a flow-relevant stenosis was to be assumed or not and documented as “present” or “absent” based on the respective combination of the PSV and flow profile.

*Treadmill examination*

Treadmill testing was performed to determine the actual current walking distance in meters. The examination took place under standard conditions with a velocity of 3 km/h and an incline of 12 %. In cases of compromised walking abilities, the settings could be adjusted by lowering the velocity in steps of 0,5 km/h or dispensing with the incline. The walking distance served as an estimation of the severity of PAD as the study subgroups were formed based on the Fontaine classification for PAD, where the walking distance is decisive. During the examination, at least one supervisor (A.T. or J.G.) was always present. All participants were asked to immediately report any pain they experienced while walking, but to continue beyond until the pain was restrictive. The walking distance until the first onset of pain was obtained and documented in the case report file (CRF) as “painless walking distance [m]”. The maximum possible walking distance was documented as “maximum walking distance [m]”. If the walking distance exceeded 500 meters, the examination was terminated due to negligible relevance, since 200 meters are of importance to clarify the PAD stage according to Fontaine[6].

*Clinical staging*

Based on the clinical test results and on the Fontaine classification for PAD[6], three merged PAD classes, including healthy volunteers, were formed for all study-related analyses. See Table S 3 for a detailed description.

*Blinding*

The examiners (A.T., J.G.) were blinded towards the results of previous reference test results during MSOT/US-measurement, the gray scale analysis of the US-images and the data evaluation. Due to obvious wounds or walking restrictions that may define the clinical PAD stage, blinding was not present in all cases. Blinding of the study participants was not necessary since no interventions were planned.

**Phantom experiments**

In order to image signal dependency with regard to muscle tissue mass/concentration, a custom-made mold was designed using Autodesk Fusion 360 (V2.0.14567, Autodesk GmbH, München, Germany) and printed with a 3D printer (Form 2, Formlabs. Inc, Somerville, MA, USA) using White Resin V4 (Formlabs. Inc, Somerville, MA, USA) [51]. The mold was filled with 2% Agarose (Biozym LE Agarose, Biozym Scientific GmbH, Hessisch Oldendorf, Germany) dissolved in distillated water. This resulted in an agarose phantom with the body dimension of 96*46*40mm and a cover of 96*46*10mm. The cover seals a recess of 76*26*20 mm, which was filled with fresh tissue solutions. This comprised of commercially available ground beef, which was diluted with deionized water in increments of 8g (20%) starting at 40g (=100%). For all experiments the MSOT imaging probe (MSOT Acuity CE, iThera Medical, München, Germany) was mounted in a laboratory bracket and coupled to the agarose phantom using transparent ultrasound gel (Aquasonic Clear, MDSS GmbH, Hannover, Germany). All measurement were repeated in triplicates.

**Statistical analysis**

*Handling of missing data*

For patients in whom MSOT/RUCT measurement of one leg was either not possible due to physical conditions (e.g., massive edema, erysipelas) or the measurement was not usable, the respective leg was excluded from the study. In such cases, if the target leg was involved, the subject was treated as a whole dropout. In case of single missing MSOT parameters, based on technical reasons, only the single values were excluded from the respective sub analyses. Missing data in one of the applied reference standards were handled through excluding the subject from the corresponding sub-analysis. Missing data occurred, for example, because an ABI could not be collected completely due to the presence of wounds or because of pain, or because the treadmill test could not be performed due to pain or other walking disabilities. A missing angiography of the nontarget leg was tolerated, but in cases where a complete angiography of the target leg was not available, the subject was excluded from the study due to not meeting all inclusion criteria.

Results

**Assessment of SWL-OAI signals during skeletal muscle degeneration**

The raised question of whether skeletal muscle mass and/or muscular degeneration is responsible for this loss of signal at 800nm. At 800nm, the absorption of HbO_2_ and HbR is found to be equal, which explains the fact that the signal might be less variable with changing oxygenation (**Figure S2A**).

To determine the contribution of muscle to the 800nm signal, a phantom experiment was performed. Therefore, custom-made molds were 3D-printed and used for imaging of muscle tissue (derived from commercial ground beef) using the identical clinical US/OAI device with identical presets (**Figure S2B**). We could show, that depending on the mass/concentration (and associated myoglobin content) the OAI signal visibly decreases in a dose-depended manner (**Figure S2C**). The quantitative SWL 800nm, HbO_2_ and HbR signal demonstrated a dose-dependent/muscle mass-dependent decrease. In turn, mSO2 remained unchanged when muscle tissue was present (**Figure S2D)**.

Supplementary Figures

**Figure S1 Dependence of SWL 800nm and GSL on depth and gender**

**A** Correlation of depth and single wavelength 800nm.

**B** Correlation of depth and gray scale level (GSL).

**C** Gender difference of single wavelength 800nm.

**D** C Gender difference of GSL.

Dots represent individual datapoints, tested with unpaired t-test.

***
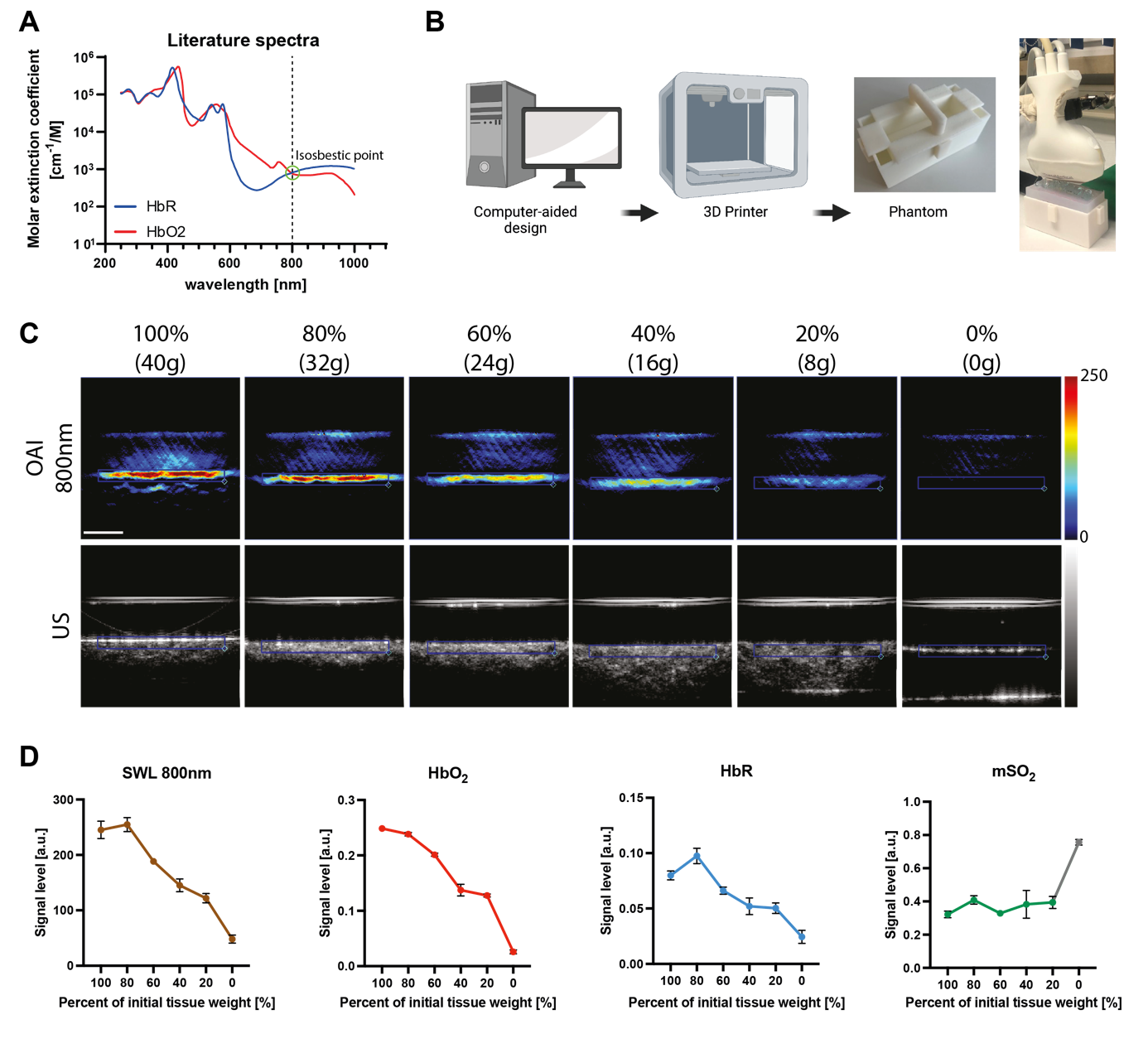
*Figure S2 Results of calf muscle biopsy sampling**

**A** Deoxygenated and oxygenated hemoglobin and their respective molecular exciton coefficients in the near infrared range of light. Spectra derived from [https://omlc.org](https://omlc.org/) and [13]; last accessed 12/01/2022. Molecular structure of ICG derived from [http://molview.org](http://molview.org/) on 19/01/2023.

**B** Development of a custom-made 3D-printed phantom. Created with BioRender.com.

**C** Representative titrated muscle phantom scans. Upper represents optoacoustic imaging signals at 800nm. Lower row represents corresponding US signals.

**D** Quantitative SWL 800nm and spectral unmixed deoxygenated hemoglobin (HbR), oxygenated hemoglobin (HbO_2_) and multispectral unmixing derived oxygenation (mSO_2_) signals over different muscle phantom masses/concentrations.

Supplementary Tables

**Table S1 Fontaine classification [6]**

| Grade | Symptoms |
| --- | --- |
| Stage I | Asymptomatic, incomplete blood vessel obstruction |
| Stage II  IIa  IIb | Mild claudication pain in limb  Claudication at a distance > 200 m  Claudication at a distance < 200 m |
| Stage III | Rest pain, mostly in the feet |
| Stage IV | Necrosis and/or gangrene of the limb |

**Table S2 Rutherford classification for chronic limb ischemia [7]**

AP: ankle pressure; PV: pulse volume recording; TM: transmetatarsal; TP: toe pressure

| Category | Clinical description | Objective criteria |
| --- | --- | --- |
| 0 | Asymptomatic – no hemodynamically significant occlusive disease | Normal treadmill or reactive hyperemia test |
| 1 | Mild claudication | Completes treadmill exercise; AP after exercise > 50 mm Hg but at least 20 mm Hg lower than resting value |
| 2 | Moderate claudication | Between categories 1 and 3 |
| 3 | Moderate claudication | Cannot complete standard treadmill exercise, and AP after exercise < 50 mm Hg |
| 4 | Ischemic rest pain | Resting AP < 40 mm Hg, flat or barely pulsatile ankle or metatarsal PVR; TP < 30 mm Hg |
| 5 | Minor tissue loss – nonhealing foot ulcer, focal gangrene with diffuse pedal ischemia | Resting AP < 60 mm Hg, ankle or metatarsal PVR flat or barely pulsatile; TP < 40 mm Hg |
| 6 | Major tissue loss – extending above TM level, functional foot no longer salvageable | Same as category 5 |

**Table S3 3-level stratified clinical (Fontaine) classification**

| Stage | Definition |
| --- | --- |
| HV | Healthy volunteer  No previously known PAD or PAD-typical symptoms, ABI 1.0-1.3, palpable foot pulses |
| IC | Intermittent claudication  Fontaine IIa and IIb; intermittent claudication at any walking distance |
| CLTI | Chronic limb threatening ischemia  Fontaine III and IV; ischemic rest pain, ulceration or gangrene |

HV: healthy volunteer; IC: intermittent claudication; CLTI: chronic limb threatening ischemia; PAD: peripheral artery disease; ABI: ankle-brachial index

**Table S4 Definition of aggregated TASC (aTASC) score based on the TASC II findings of aortoiliac (AI), femoropopliteal (FP), and infrapopliteal (IP) vessel sections**

|  | aTASC 1 | aTASC 2 | aTASC 3 |
| --- | --- | --- | --- |
| Description | included as HV with normal clinical findings or no arteriosclerotic findings in angiography | assumption of sufficient collateralization due to mild findings in AI or FP section | assumption of poor collateralization due to severe AI findings |
| Combination of TASC II findings | 1. no findings in AI TASC  2. no findings in FP TASC  3. no findings in IP TASC | 1. no or A/B findings in AI TASC  2. no or A/B/C/D findings in FP TASC  3. any findings in IP TASC  4. at least TASC A/B in 1. or 2. | 1. C/D findings in AI TASC  2. no or A/B/C/D findings in FP TASC  3. any findings in IP TASC |

TASC: Trans-Atlantic Inter-Society Consensus; PAD: Peripheral Artery Disease; CIA: common iliac artery; EIA: external iliac artery; CFA: common femoral artery; AAA: abdominal aortic aneurysm

TASC II classifications for aortoiliac (AI) PAD: Type A: unilateral/bilateral stenoses of CIA or uni-/bilateral single short (< 3 cm) stenosis of EIA; Type B: short (> 3 cm) stenosis of infrarenal aorta or unilateral CIA occlusion or single or multiple stenosis totaling 3-10 cm involving the EIA not extending into the CFA or unilateral EIA occlusion not involving the origins of internal iliac or CFA; Type C: bilateral CIA occlusions or bilateral EIA stenoses 3-10 cm long not extending into the CFA or unilateral EIA stenosis extending into the CFA or unilateral EIA occlusions that involves the origins of internal iliac and/or CFA or heavily calcified unilateral EIA occlusion with or without involvement of origins of internal iliac and/or CFA; Type D: Infrarenal aortoiliac occlusion or diffuse disease involving the aorta and both iliac arteries requiring treatment or diffuse multiple stenoses involving the unilateral CIA, EIA, and CFA or unilateral occlusions of both CIA and EIA or bilateral occlusions of EIA or iliac stenoses in patients with AAA requiring treatment and not amendable to endograft placement or other lesions requiring open aortic or iliac surgery

TASC II classifications for femoropopliteal (FP) PAD: Type A: Single stenosis ≤ 10 cm in length or single occlusion ≤ 5 cm in length; Type B: Multiple lesions (stenoses or occlusions), each ≤ 5 cm or single stenosis or occlusion ≤ 15 cm not involving the infrageniculate popliteal artery or single or multiple lesions in the absence of continuous tibial vessels to improve inflow for a distal bypass or heavily calcified occlusions ≤ 5 cm in length or single popliteal stenosis; Type C: Multiple stenoses or occlusions totaling ≥ 15 cm with or without heavy calcification or recurrent stenoses or occlusions that need treatment after two endovascular interventions; Type D: Chronic total occlusion of CFA or SFA (> 20 cm, involving the popliteal artery) or chronic total occlusion of popliteal artery and proximal trifurcation vessels,

TASC II classification for infrapopliteal (IP) PAD: Type A: Single focal stenosis, < 5 cm in length, in the target tibial artery with occlusion or stenosis of similar or worse severity in the other tibial arteries; Type B: Multiple stenoses, each ≤ 5 cm in length, or total length ≤ 10 cm or single occlusion ≤ 3 cm in length, in the target tibial artery with occlusion or stenosis of similar or worse severity in the other tibial arteries; Type C: Multiple stenoses in the target tibial artery and/or single occlusion with total lesion length > 10 cm with occlusion or stenosis of similar or worse severity in the other tibial arteries; Type D: Multiple occlusions involving the target tibial artery with total lesion length > 10 cm or dense lesion calcification or non-visualization of collaterals. The other tibial arteries occluded or dense calcification

**Table S5 Multiple logistic regression for clinical PAD stages**

| **Factor** | **AUC** | **95%CI** | **Sensitivity (%)** | **Specificity (%)** | **P-Value** |
| --- | --- | --- | --- | --- | --- |
| Depth | 0.53 | 0.42-0.64 | 20.7 | 91.5 | 0.5745 |
| Gender | 0.61 | 0.51-0.71 | 70.7 | 50.9 | 0.0446 |
| 800nm | 0.64 | 0.54-0.74 | 6.9 | 98.3 | 0.0083 |
| GSL | 0.74 | 0.65-0.83 | 20.7 | 98.3 | <0.0001 |
| Age | 0.80 | 0.72-0.87 | 25.8 | 98.3 | <0.0001 |
| GSL+800nm | 0.80 | 0.71-0.88 | 70.7 | 76.3 | <0.0001 |
| GSL+800nm+gender | 0.81 | 0.73-0.89 | 83.3 | 70.7 | <0.0001 |
| 800nnm+age | 0.81 | 0.73-0.89 | 70.7 | 69.5 | <0.0001 |
| GSL+age | 0.85 | 0.78-0.92 | 77.6 | 79.7 | <0.0001 |
| GSL+800nm+age | 0.88 | 0.81-0.94 | 82.8 | 76.3 | <0.0001 |
| GSL+800nm+gender+age+depth | 0.88 | 0.82-0.94 | 81.0 | 79.7 | <0.0001 |

AUC: area-under-the-curve, 95%CI: 95% confidence interval, GSL: gray scale level

**Table S6 Multiple logistic regression for angiographic PAD stages**

| **Factor** | **AUC** | **95%CI** | **Sensitivity (%)** | **Specificity (%)** | **P-Value** |
| --- | --- | --- | --- | --- | --- |
| Depth | 0.58 | 0.43-0.73 | 5.6 | 98.3 | 0.3135 |
| Gender | 0.64 | 0.50-0.78 | 77.8 | 50.9 | 0.0765 |
| Age | 0.70 | 0.57-0.82 | 5.6 | 96.6 | 0.0123 |
| 800nm | 0.70 | 0.57-0.84 | 11.1 | 100 | 0.0093 |
| 800nnm+age | 0.74 | 0.62-0.87 | 16.7 | 94.8 | 0.0018 |
| GSL | 0.77 | 0.63-0.90 | 33.3 | 94.8 | 0.0007 |
| GSL+age | 0.82 | 0.71-0.93 | 44.4 | 93.1 | <0.0001 |
| GSL+800nm | 0.83 | 0.72-0.94 | 50.0 | 93.2 | <0.0001 |
| GSL+800nm+Age | 0.84 | 0.74-0.95 | 61.1 | 93.1 | <0.0001 |
| GSL+800nm+Gender | 0.85 | 0.76-0.95 | 50.0 | 91.4 | <0.0001 |
| GSL+800nm+Gender+Age+Depth | 0.86 | 0.76-0.96 | 61.1 | 93.1 | <0.0001 |

AUC: area-under-the-curve, 95%CI: 95% confidence interval, GSL: gray scale level

**Table S7 Categories for ankle-brachial index (ABI) [4]**

| Category | ABI value |
| --- | --- |
| No PAD | 1.0-1.3 |
| Mild PAD | 0.75-0.9 |
| Moderate PAD | 0.5-0.74 |
| Severe PAD | <0.5 |
| Mediasclerosis | >1.3 |

PAD: peripheral artery disease

References

1. Schindelin, J., et al., Fiji: an open-source platform for biological-image analysis. Nature Methods, 2012. **9**(7): p. 676-82.

2. Regensburger, A.P., et al., Detection of collagens by multispectral optoacoustic tomography as an imaging biomarker for Duchenne muscular dystrophy. Nature Medicine, 2019. **25**: p. 1905-1915.

3. Prockop, D.J. and S. Udenfriend, A specific method for the analysis of hydroxyproline in tissues and urine. Anal Biochem, 1960. **1**: p. 228-39.

4. Huppert, P., J. Tacke, and H. Lawall, S3-Leitlinien zur Diagnostik und Therapie der peripheren arteriellen Verschlusskrankheit. Der Radiologe, 2010. **50**: p. 7-15.

5. Aboyans, V., et al., 2017 ESC Guidelines on the Diagnosis and Treatment of Peripheral Arterial Diseases, in collaboration with the European Society for Vascular Surgery (ESVS). European Heart Journal, 2018. **39**: p. 763-821.

6. Fontaine, R., M. Kim, and R. Kieny, [Surgical treatment of peripheral circulation disorders]. Helv Chir Acta, 1954. **21**(5-6): p. 499-533.

7. Hardman, R.L., et al., Overview of classification systems in peripheral artery disease. Seminars in Interventional Radiology, 2014. **31**(4): p. 378-88.
